# Risk factors for bacteremia in adult febrile patients in emergency settings

**DOI:** 10.1186/cc14091

**Published:** 2015-03-16

**Authors:** A Mikami, Y Natori, F Omata, S Ishimatsu

**Affiliations:** 1 St Luke's International Hospital, Tokyo, Japan; 2 St Luke's Life Science Institute, Tokyo, Japan

## Introduction

Blood culture is a critical procedure for detecting potentially life-threatening bloodstream infections (BSI). At the same time, early diagnosis and appropriate treatment of BSI are the key factors in order to improve prognosis. The purpose of the current analysis was to identify risk factors for bacteremia in adult febrile patients in emergency settings.

## Methods

We conducted a retrospective case-control study within a population of adult patients visiting the emergency department at a community hospital (St Luke's International Hospital, Tokyo, Japan) and who underwent two sets of blood culture testing between 2003 and 2012. Among a total of 13,582 patients, 1,322 (10%) were detected as bacteremia. We included in this study 179 randomly selected patients from the bacteremia group and 321 randomly selected patients from the negative blood culture group to serve as the comparison group. Multivariate logistic regression was used to evaluate the relationship between clinical characteristics factors and bacteremia.

## Results

In a multivariate logistic regression model, a statistically significant independent effect was found for body temperature (BT) >38°C (OR = 2.58, 95% CI, 1.76 to 3.79, *P *< 0.001), systolic blood pressure (SBP) <100 mmHg (OR = 1.72, 95% CI, 1.11 to 2.65, *P *= 0.01), CRP >10 mg/dl (OR = 3.03, 95% CI, 2.05 to 4.49, *P *< 0.001) and PaCO_2 _<32 mmHg (OR = 2.3, 95% CI, 1.57 to 3.37, *P *< 0.001). Receiver operating characteristic curve analysis revealed an area under the curve value of 0.725 for differentiating patients with bacteremia from negative culture.

## Conclusion

BT >38°C, SBP <100 mmHg, CRP >10 mg/dl and PaCO_2 _<32 mmHg are independently associated with bacteremia. These factors might be useful to know whether or not adult febrile patients have bacteremia.
